# GM-CSF Promotes Superior In Vitro Differentiation of MHC II^+^ CD11c^+^ APCs Compared to L-929 Supernatant in Mouse Bone Marrow Cultures

**DOI:** 10.3390/ijms27104637

**Published:** 2026-05-21

**Authors:** Gabriel Cordeiro, Felipe Cezar Pinheiro de Mato, Amanda Pires Bonfanti, Liana Verinaud, Catarina Rapôso

**Affiliations:** 1 Laboratory of Immunopatologies, Departament of Microbiology and Immunology, Institute of Biology, Universidade Estadual de Campinas, Campinas 13083-970, Brazil; gabrielcordeiro.pr@gmail.com (G.C.); felipecezar_pinheiro@hotmail.com (F.C.P.d.M.); amanda_bonfanti@hotmail.com (A.P.B.); verinaud@unicamp.br (L.V.); 2 Laboratory of Advanced Theraphies, Faculty of Pharmaceutical Sciences, Universidade Estadual de Campinas, Campinas 13083-862, Brazil

**Keywords:** protocol comparison, stem cell, dendritic cell, macrophage, C57BL/6J

## Abstract

Antigen-presenting cells (APCs) play a critical role in modulating immune responses, making the optimization of their differentiation protocols essential for advancing cell-based immunotherapies. This study evaluated eight protocols to differentiate APCs from bone marrow precursors of C57BL/6J mice, comparing the effects of GM-CSF and L-929 conditioned supernatants at various concentrations. Four groups treated with GM-CSF and four with L-929 supernatant, alongside a control group, were assessed. Flow cytometry analysis revealed that GM-CSF significantly increased the yield of CD11c^+^ MHC II^+^ cells by up to 6-fold compared to the L-929 supernatant. Furthermore, GM-CSF-treated groups showed higher mean fluorescence intensities (MFI) for critical markers such as MHC II and CD11c, with MFI levels surpassing those of SL-929-treated groups by approximately 3- to 5-fold. In contrast, the L-929 supernatant demonstrated limited efficacy in promoting both cell differentiation and surface marker expression, resulting in minimal phenotypic and quantitative gains compared to controls. These findings highlight the superior efficiency of GM-CSF in driving APC differentiation and underscore the importance of balancing cell yield and phenotypic fidelity when selecting differentiation protocols. This study provides valuable insights for researchers developing targeted immunotherapies and offers a solid foundation for optimizing APC-dependent therapies, ensuring efficacy and cost-efficiency in cell-based strategies.

## 1. Introduction

When the body is threatened by pathogens, or even by cellular and non-cellular components of the host itself, antigen-presenting cells (APC) are responsible for initiating the adaptive response. Monocytes, macrophages, and dendritic cells (DCs), members of this group, are known to express various surface proteins used as molecular markers for identification. These APCs are critical for the processing and presentation of antigens to T cells, thereby orchestrating immune responses. Surface proteins such as MHC II, CD11c, CD11b, and F4/80 play a significant role in the functionality of these populations, and the expression of some of them is used as a parameter to validate the quality of therapies such as cell-based. These markers not only define cellular identity but also reflect functional capabilities, such as antigen uptake, processing, and presentation, which are essential for activating T cells and orchestrating immune responses [1,2]. For instance, CD11c and MHC II are indispensable for antigen presentation and T-cell activation, playing a pivotal role in immune modulation.

APC-dependent cell therapy is emerging as a promising therapeutic avenue for diverse pathologies, including cancer, autoimmune disorders, and infectious diseases, highlighting the crucial dependence on protocols to elicit or differentiate specific cell populations. This promise is reflected in ongoing clinical trials targeting the treatment of several diseases, paving the way for new therapeutic options for patients [3,4,5,6,7]. This therapeutic approach leverages the inherent antigen processing and presentation capabilities of APCs, predominantly DCs, to orchestrate a targeted immune response against specific pathogens or tumor cells. DC-based therapies have been explored as a strategy to modulate immune responses, with applications aimed at either inducing antigen-specific immunity, such as in cancer treatments, or promoting tolerance in autoimmune and inflammatory conditions [8,9]. However, the success of immunostimulatory DC-based therapies depends on multiple factors, including efficient DC migration, antigen uptake and processing, and the presence of an activating microenvironment to ensure proper T-cell activation. Optimizing these parameters is critical, and ongoing studies are investigating ways to improve APC-based immunotherapy through in vitro maturation, antigen loading, cytokine stimulation, and pharmacological modulation, followed by targeted delivery near lymph nodes in the tumor region [10,11,12,13]. High cost and time consumption in the development of APC therapies are some of the problems that limit progress in this field. Therefore, it is necessary that new protocols be developed to optimize and make better use of available resources.

In the bone marrow, hematopoietic stem cells (HSCs) result in several cell types, including DCs and macrophages [14]. It is already known that cellular differentiation can be directly influenced by various chemical and physical factors, such as Colony-Stimulating Factors (CSFs), temperature, and gravity [15,16,17]. There are several methods to differentiate APCs from HSCs in vitro. The use of a Macrophage Colony-Stimulating Factor (M-CSF) and a Granulocyte–Monocyte Colony-Stimulating Factor (GM-CSF) are the most common approaches, and their concentrations are also known to influence the type and percentage of differentiated cells [18,19,20]. GM-CSF is the most commonly used cytokine to differentiate DCs and macrophages, and the concentration of this substance in the medium can directly influence the cell type and function [21]. Furthermore, some protocols indicate a conditioned medium with the L-929 supernatant (SL-929) for macrophage differentiation [22,23]. L-929, a fibroblast cell line, is known to secrete GM-CSF and M-CSF at different concentrations. However, there is currently no specific protocol using the L-929 supernatant that demonstrates the same effectiveness for APC profiles other than macrophages.

Understanding the phenotypic profile of these cells is critical to assess their functional capabilities. Refining the protocol using both SL-929 and GM-CSF may be important to aid in the development of research that requires more specific cell profiles. With the aim of assisting in the development of new APC-dependent therapies and optimizing the use of resources, this study investigates the impact of different concentrations of GM-CSF and SL-929 on the differentiation of progenitor cells into distinct APC populations. We hypothesize that variations in the concentrations of GM-CSF and SL-929 influence the generated cellular profiles and can be optimized for therapeutic applications.

## 2. Results

### 2.1. GM-CSF Increases the Number of Differentiated Cells Better than SL-929

**Figure 1:**
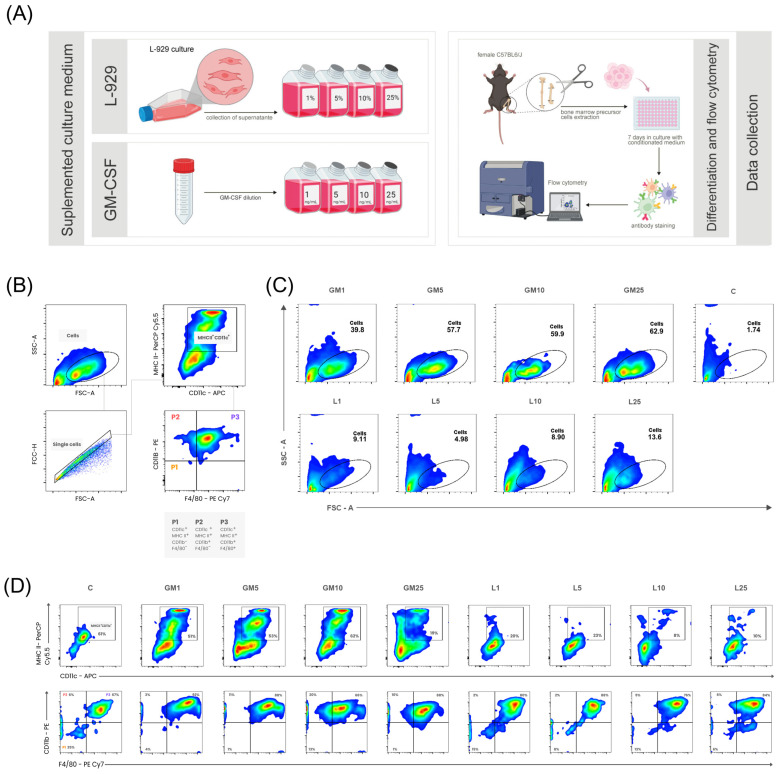
(**A**) Schematic representation of the experimental methodology. The upper panel shows the preparation of SL-929 conditioned medium at 1%, 5%, 10%, and 25% concentrations. The lower panel shows GM-CSF supplemented medium at 1, 5, 10, and 25 ng/mL. Bone marrow precursor cells from C57BL/6J mice were cultured for 7 days in these mediums, followed by antibody staining and flow cytometry analysis. (**B**) Gating strategy: Thresholds for forward scatter (FSC) and side scatter (SSC) were applied to exclude debris and artifacts, ensuring that the analysis was restricted to intact cells; doublets were excluded using forward-scatter-height versus forward-scatter-area (FSC-H vs. FSC-A) plots. APCs were identified based on the co-expression of MHC II and CD11c markers and further stratified into three subpopulations: P1 (MHC II^+^ CD11c^+^ CD11b^−^ F4/80^−^); P2 (MHC II^+^ CD11c^+^ CD11b^+^ F4/80^−^); and P3 (MHC II^+^ CD11c^+^ CD11b^+^ F4/80^+^). (**C**) Different concentrations of GM-CSF and SL-929 differentiate bone marrow precursor cells into heterogeneous populations. Each graph shows the gated region (cells) used for identifying and analyzing subpopulations. Cells conditioned with GM-CSF exhibited higher percentages in the gate of interest compared to those conditioned with SL-929 or control. (**D**) Flow cytometry plots representing the experimental groups were analyzed using the same gating strategy established in Figure 1B. MHC II⁺ CD11c⁺ cells were further stratified into three subpopulations: P1 (MHC II⁺ CD11c⁺ CD11b⁺ F4/80^−^), P2 (MHC II⁺ CD11c⁺ CD11b⁺ F4/80⁺), and P3 (MHC II⁺ CD11c⁺ CD11b^−^ F4/80⁺). The percentages shown in the plots are relative to the total number of events displayed in each graph. The SL-929-treated groups exhibited the lowest percentages of MHC II⁺ CD11c⁺ cells. Additionally, most MHC II⁺ CD11c⁺ cells expressed both CD11b and F4/80. Each experimental condition was performed in triplicate and repeated in two independent experiments, resulting in a total of six replicates per group (*n* = 6). C—control group; GM—GM-CSF groups; L—SL-929 groups.

To investigate the effects of GM-CSF and SL-929 on the differentiation of bone marrow-derived cells, a comparative study was conducted using eight distinct culture media, including four concentrations of GM-CSF (1, 5, 10, and 25 ng/mL) and the supernatant from the L-929 cell culture (1%, 5%, 10%, and 25%). Bone marrow cells from C57BL/6J mice were cultured for 7 days in the presence of these media. After the culture period, cell morphology was assessed by light microscopy, and phenotypic analysis was performed by flow cytometry using specific antibodies against CD11c, MHC II, CD11b, and F4/80. The experimental workflow is illustrated in Figure 1A.

Three distinct populations were identified based on surface marker expression (Figure 1B). The gating strategy involved an initial selection of MHC II^+^ CD11c^+^ cells, followed by subsequent gating to identify CD11b^−^ F4/80^−^ (P1), CD11b^+^ F4/80^−^ (P2), and CD11b^+^ F4/80^+^ (P3) populations. These populations were classified as APCs based on their expression of MHC II and CD11c, which are commonly used markers for dendritic cells and other APC subsets, though not exclusively to all antigen-presenting cells. Although a precise functional characterization of these populations was beyond the scope of this study, the marker profiles suggest that P1, P2, and P3 likely correspond to conventional dendritic cells type 1 (cDC1), type 2 (cDC2), and monocyte-derived dendritic cells (moDCs), respectively, based on the unique expression patterns of their surface markers. While discussing these populations, it is essential to note that P1 is characterized by the absence of CD11b and F4/80, a profile that aligns with the phenotypic characteristics of cDC1. In contrast, P2 displays a CD11b^+^ but F4/80^−^ profile, which corresponds to cDC2, while P3 demonstrates coexpression of CD11b and F4/80, indicative of moDCs. This classification was derived following an initial gating of MHC II^+^ CD11c^+^ cells, subsequently segregating the populations based on CD11b and F4/80 expression, ensuring a logical separation of the identified subgroups.

Our results indicate a significant increase in the number of cells within the target populations after GM-CSF stimulation (Figure 1C). This effect was concentration-dependent, with the highest concentration (GM25) yielding the largest proportion of cells (62.9%), followed by GM10 (59.9%), GM5 (57.7%), and GM1 (39.8%). In contrast, SL-929 treatment resulted in a modest increase in cell numbers compared to the control group (1.74%). Among the SL-929-treated groups, the highest percentage of cells was observed at L25 (13.6%), followed by L10 (8.9%), L1 (9.11%), and L5 (4.98%).

Flow cytometry plots of the experimental groups, analyzed using the same gating strategy as in Figure 1B (Figure 1D), reveal MHC II^+^ CD11c^+^ cells. These cells were further categorized into P1, P2, and P3 populations based on CD11b and F4/80 expression, allowing for a more refined characterization of antigen-presenting cells. Notably, GM-CSF-treated groups exhibited the highest proportions of MHC II^+^ CD11c^+^ CD11b^+^ F4/80^+^ cells, with this effect being particularly pronounced at higher concentrations (GM10 and GM25). This result suggests a concentration-dependent influence of GM-CSF on the differentiation and expansion of specific APC subsets.

**Figure 2:**
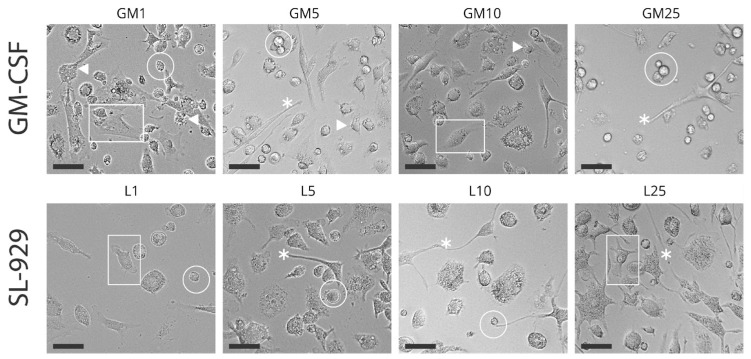
Morphological diversity in cell populations after differentiation under GM-CSF and SL-929 conditions at day 7. Cells exhibit varied morphologies, including a spectrum of large and small, round-shape cells, adherent and semi-adherent phenotypes, dendritic projections, and intracellular granularity. These features are observed at different concentrations of GM-CSF (1, 5, 10, and 25 ng/mL) and SL-929 (1%, 5%, 10%, and 25%). Notably, larger, adherent cells with intracellular granularity and elongated cytoplasmic processes are consistent with P3, whereas smaller, dendritic-shaped cells are more representative of P1 and P2. Undifferentiated monocytes-like morphology was observed more frequently in groups treated with 25 ng/mL GM-CSF and 1% SL-929, demonstrating that these conditions were less effective in promoting differentiation, particularly of P3. Arrowheads indicate cells with dendritic morphology, circles highlight nonadherent cells, rectangles denote adherent cells, and asterisks mark cells with long cellular projections. C—control group; GM—GM-CSF groups; L—SL-929 groups. Scale bars, 50 μm.

In addition to quantitative differences, morphological analysis revealed distinct phenotypic characteristics between the treatment groups (Figure 2). GM-CSF-treated cultures predominantly exhibited larger, adherent cells with visible dendritic projections and elongated cytoplasmic processes, features indicative of advanced differentiation, particularly within the P3 population. In contrast, cells cultured with SL-929 displayed a more rounded morphology, suggesting a less differentiated state. These morphological findings align with the quantitative data, further supporting the superior ability of GM-CSF to enhance both the differentiation and expansion of antigen-presenting cell populations in vitro.

### 2.2. GM-CSF Improves APC Differentiation and Marker Expression Compared to SL-929

**Figure 3:**
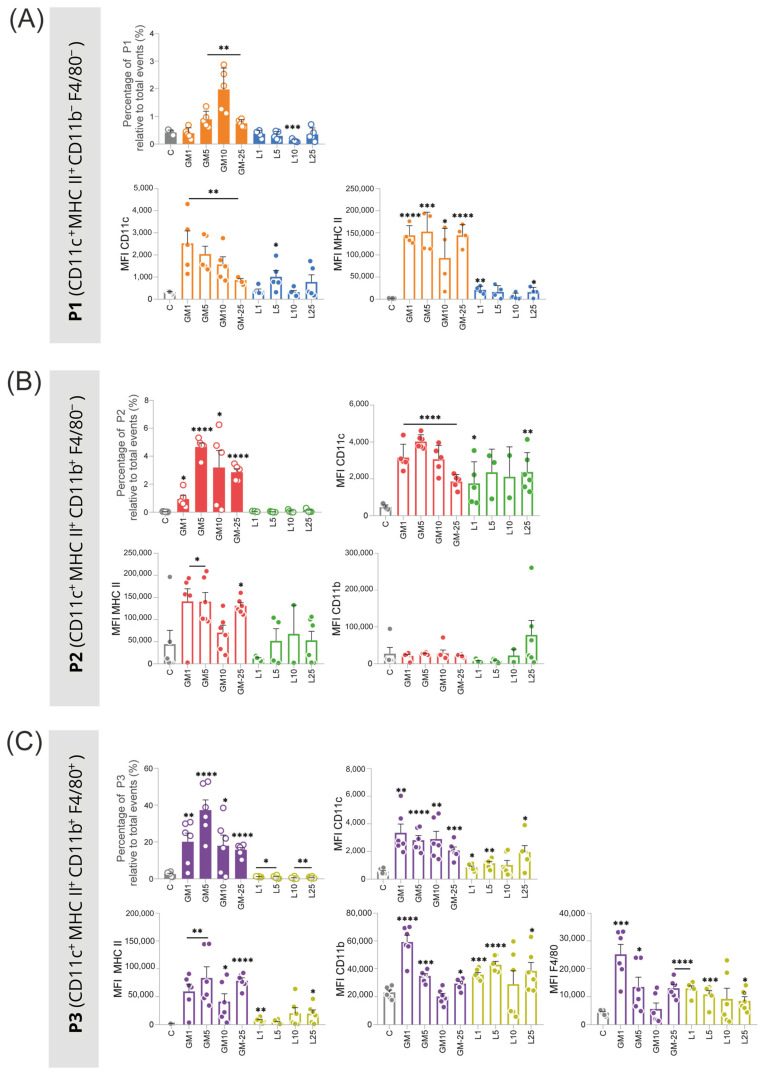
Number of cells and mean fluorescence intensity (MFI) of surface proteins from the three populations of interest acquired by flow cytometry. (**A**) The P1 population (CD11c^+^ MHC II^+^ CD11b^−^) was favored by GM-CSF (10 ng/mL), showing up to six times higher levels of CD11c and MHC II surface proteins compared to cells conditioned with SL-929. (**B**) The P2 population (CD11c^+^ MHC II^+^ CD11b^+^) exhibited the lowest percentage with GM-CSF at 1 ng/mL, but this effect was not concentration-dependent, as 5 ng/mL resulted in the highest percentage. (**C**) The P3 population (CD11c^+^ MHC II^+^ CD11b^+^ F4/80^+^) was the most predominant. GM-CSF at 5 ng/mL was the most effective for this profile. SL-929 stimulated MHC II expression but less effectively than GM-CSF. In all populations, SL-929 did not significantly increase the number of differentiated cells, similar to the unconditioned control group. These findings suggest that GM-CSF is more effective in promoting the differentiation of APCs, highlighting the importance of optimizing culture conditions for specific therapeutic applications. Statistical significance was determined using *t*-tests comparing each treatment group to the control. Each experimental condition was performed in triplicate and repeated in two independent experiments, resulting in a total of six replicates per group (*n* = 6). C—control group; GM—GM-CSF groups; L—SL-929 groups; MFI—mean fluorescence intensity. Data are presented as mean ± SEM. Statistical significance was determined using unpaired Student’s *t*-tests, comparing each treatment group to the control. A *p*-value < 0.05 was considered statistically significant. Significance levels are indicated as follows: * *p* < 0.05, ** *p* < 0.01, *** *p* < 0.001, **** *p* < 0.0001.

GM-CSF stimulation significantly increased the differentiation of APC populations, with the highest yield of differentiated cells observed in P1 at 10 ng/mL, where the percentage of cells was at least 2-fold higher than in other conditions (Figure 3A). Concentrations of 5, 10, and 25 ng/mL yielded the highest number of cells in P2 (Figure 3B). For P3, all concentrations promoted an increase in the percentage of cells, with 5 ng/mL being the most effective in promoting the proliferation of this population (Figure 3C).

In addition to promoting differentiation, GM-CSF significantly enhanced the expression of CD11c in all populations. CD11c expression levels were consistently higher in GM-CSF-treated cells compared to other conditions, indicating a strong effect of GM-CSF in upregulating this critical APC marker. Furthermore, GM-CSF was enhanced.

MHC II was expressed across all populations, with the most pronounced effects observed at concentrations of 1 ng/mL, 5 ng/mL, and 25 ng/mL.

In contrast, SL-929 was significantly less effective in promoting both cell differentiation and surface marker expression. Cells treated with SL-929 showed markedly lower levels of CD11c and MHC II expression compared to those treated with GM-CSF, with minimal differentiation and marker upregulation observed. These results underscore the superior efficacy of GM-CSF in driving APC differentiation and the upregulation of critical APC markers compared to SL-929.

## 3. Discussion

In this study, we identified three distinct populations following in vitro differentiation of murine bone marrow precursor cells using GM-CSF and SL-929: P1 cells (CD11c^+^, MHC II^+^, CD11b^−^, F4/80^−^), P2 cells (CD11c^+^, MHC II^+^, CD11b^+^, F4/80^−^), and P3 cells (CD11c^+^, MHC II^+^, CD11b^−^, F4/80^+^). It is important to note that in human cell-based therapies generalist markers such as MHC II, CD11c, and CD11b, as well as their human equivalents, are routinely used to assess the purity and phenotypic identity of differentiated cell populations [24]. Based on their surface markers, we hypothesized that P1 corresponds to conventional DC type 1 (cDC1), P2 to conventional DC type 2 (cDC2), and P3 to monocyte-derived DC (moDC) [25,26]. Additionally, the coexpression of markers such as CD11c, MHC II, CD11b, and F4/80 suggests that some populations may overlap with macrophage phenotypes, highlighting the plasticity of APCs and their potential for functional diversity. Although our classification aligns with previously established criteria, we acknowledge that additional markers such as XCR1, CD1α, CD64, and CD172a (SIRPα) would provide a more precise identification of these subsets. Future studies should incorporate these markers to refine the phenotypic characterization of these populations. In addition to phenotypic characterization, functional validation remains essential to confirm the antigen-presenting capacity of these cells.

Since these cultures originate from bone marrow progenitors, a diverse range of myeloid cells is expected. However, our study was designed to evaluate APC differentiation efficiency rather than providing a full hematopoietic characterization. Future studies focusing on the functional role of non-APC populations in these cultures may provide additional insights into the cellular interactions influencing APC development. The most abundant population observed in our cultures corresponded to a moDC-like phenotype. Interestingly, the coexpression of CD11b and F4/80 in P3, while typically associated with macrophages, has also been documented in moDCs [27]. Previous studies have established that a concentration of 10 ng/mL GM-CSF yields the most favorable population ratio of dendritic cells (DCs) aftetr BMPC differentiation [15]. In the present study, we demonstrated that a concentration of 5 ng/mL GM-CSF is also effective, with this effect being most prominent in the differentiation of moDC profile, which may contribute to reducing production costs. Furthermore, this population exhibited variations in the mean fluorescence intensity (MFI) of key markers, such as MHC II and CD11c, which appeared to be influenced by the specific conditions applied. It is noteworthy that reduced surface expression of MHC II and CD11c may compromise the antigen-presenting capacity and overall functional performance of DCs [28,29,30].

The superior differentiation of cell populations observed with GM-CSF compared to SL-929 is likely attributed to differences in the levels of colony-stimulating factors (CSFs) within the medium. As reported by Englen et al. [31], GM-CSF concentrations in SL-929 supernatant are relatively low, which may limit its efficacy in promoting APC differentiation. Furthermore, L-929 cells are known to secrete over 2500 proteins [22], many of which may influence the differentiation process through mechanisms that remain to be fully elucidated. The L-929 supernatant is widely recognized as a source of CSFs, particularly the macrophage colony-stimulating factor (M-CSF), and is commonly employed for the differentiation of macrophages from bone marrow precursors. Studies have demonstrated that a medium conditioned with 10% L-929 supernatant effectively induces macrophages exhibiting an inflammatory profile comparable to those differentiated with M-CSF while presenting a distinct metabolic signature [32]. Nevertheless, its ability to generate antigen-presenting cells with dendritic characteristics is significantly lower than that of GM-CSF. This is consistent with the findings of the present study, where L-929-treated cultures displayed a reduced expression of APC-associated markers, such as CD11c and MHC II, and produced fewer differentiated cells across all analyzed subpopulations.

The observed differences in APC differentiation efficiency between GM-CSF and SL-929 highlight the importance of cytokine concentration and composition in the culture medium. The ability of GM-CSF to consistently produce higher yields of differentiated APC cells underscores its potential as the optimal cytokine to generate cell populations required in large numbers for cell-based immunotherapies researchers. This is particularly relevant for therapeutic strategies where cell dose is a critical factor for efficacy, such as in cancer immunotherapy. Although our findings are based on murine cells, they suggest that optimizing GM-CSF concentrations may be critical in human DC differentiation protocols. These findings indicate that SL-929 is not recommended for generating the APC profiles observed in our study when high cell yields are required. Future studies should explore whether similar effects can be observed in human systems, potentially leading to more effective and personalized immunotherapy approaches.

## 4. Materials and Methods

### 4.1. Reagent and Animals

The reagents were obtained from Sigma–Aldrich (St. Louis, MO, USA), eBioscience (Waltham, MA, USA), and Cultilab (Campinas, Brazil). All experiments were conducted in accordance with the Ethical Principles on Animal Research, adopted by the Brazilian College of Animal Experimentation (Colégio Brasileiro de Experimentação Animal—COBEA), with the prior approval by the Ethics Committee on the Use of Animals (CEUA/UNICAMP—Universidade Estadual de Campinas; protocol number: 5074-1/2018). Females C57BL/6J mice, 6–8 weeks old, from the Multidisciplinary Centre for Biological Research at Universidade Estadual de Campinas, were used in this study. Mice were kept in specific-pathogen-free condition in a controlled temperature and photoperiod environment, with autoclaved food and water ad libitum throughout the experiment. The animals were kept in the Animal Facility of the Biology Institute, Department of Genetic, Evolution, Microbiology, and Immunology, UNICAMP, Campinas, São Paulo, Brazil.

### 4.2. L-929 Culture and Supernatant

First, 2 × 10^6^ L-929 cells (murine fibroblasts) were placed in 25 cm cell culture flasks. The culture was incubated with 20 mL of IMDM (Iscove’s Modified Dulbecco’s Medium) containing 10% of serum fetal bovine (SFB) and 50 mg/mL of L-glutamine, penicillin, and streptomycin solution (Gibco Inc., Billings, MT, USA) at 37 °C in an oven with 5% CO_2_. The medium was not replaced during the experiment. The supernatant was collected 2 days after total confluence. The pH was measured, and the supernatant of 10 flasks of L-929 cells was used for the differentiation of the cells. The culture medium was supplemented with L-929 supernatant at concentrations of 1%, 5%, 10%, and 25% of the total culture medium volume.

### 4.3. Generation of APCs

Bone marrow precursor cells were isolated from female C57BL/6 mice (6–8 weeks old) maintained under SPF conditions, following the protocol described by Tang et al. [33]. Femurs and tibiae were carefully dissected and cleared of surrounding muscle tissue. The bones were then briefly disinfected in 70% ethanol (2–5 min) and rinsed with PBS. To extract the bone marrow, both ends of the bones were cut with sterile scissors inside a Petri dish containing IMDM medium (Iscove’s Modified Dulbecco’s Medium—I7633/Sigma) without fetal bovine serum. The bones were held with forceps over a second Petri dish containing IMDM supplemented with fetal bovine serum (cultilab—10%, *v/v*) and antibiotic antimycotic solution (MFCD00130520/Sigma—50 mg/mL) to avoid direct contact with the solution. The marrow was then flushed out using a syringe filled with the solution of the second Petri dish and fitted with a 0.45 mm diameter needle. The resulting cell suspension was homogenized by vigorous pipetting to ensure single-cell dissociation and subsequently centrifuged at 1500 RPM for 5 min at 4 °C to remove debris and collect the cellular fraction.

Then, 2 × 10^5^ cells were seeded in 96-well culture plate containing complete medium supplemented with GM-CSF (1 ng/mL, 5 ng/mL, 10 ng/mL, or 25 ng/mL) or SL-929 (*v*/*v*: 1%, 5%, 10%, or 25%) and maintained for 7 days in culture. Fresh medium was added every 2 days of culture containing the supplementations. In the control group, cells were plated only with a complete medium without stimulus. For phase-contrast imaging, cells were plated in 35 mm Petri dishes at a density of 2 × 10^5^ cells per dish, maintaining the same conditions as those used in the 96-well plates to ensure comparability while avoiding excessive cell overlap. Live cell images were captured on day 7 of culture using a light microscope at 400× magnification. On the same day, cells were removed from the 96-well plates, centrifuged, and marked for flow cytometry. Each experimental condition was performed in triplicate and repeated in two independent experiments, resulting in a total of six replicates per group (*n* = 6).

### 4.4. Flow Cytometry

Each treatment group was individually homogenized in 1 mL of sterile phosphate-buffered saline (PBS, 0.02 M) to wash and remove the culture medium. Cell viability was assessed using Trypan Blue (Sigma). The following antibodies were used for immunostaining (all from eBioscience, San Diego, CA, USA): anti-MHC II (clone M5/114.15.2, PerCP Cy5.5, #107626), anti-CD11c (clone N418, APC, #117310), anti-CD11b (clone M1/70, PE, #557397), and anti-F4/80 (clone BM8, PE-Cy7, #25-4801-82). The cells were incubated with these antibodies for 30 min at 4 °C in the dark. After incubation, cells were fixed with 1% para-formaldehyde, washed to remove all fixative, and resuspended in PBS for analysis. Data were acquired from 20,000 events per sample using a FACSVerse flow cytometer (BD Biosciences, Herlev, Denmark), located at the Department of Genetics, Evolution, Microbiology, and Immunology, Campinas, São Paulo, Brazil) and analyzed with FlowJo v10 software (Becton Dickinson & Company, Franklin Lakes, NJ, USA).

Thresholds for forward scatter (FSC) and side scatter (SSC) were applied to exclude debris and artifacts, ensuring that the analysis focused on intact cells. Doublets were removed using forward-scatter-height versus forward-scatter-area (FSC-H vs. FSC-A) plots to retain only single-cell events. Antigen-presenting cells (APCs) were identified based on the co-expression of MHC II and CD11c markers. These cells were further stratified into three distinct subpopulations: P1 (MHC II^+^ CD11c^+^ CD11b^−^ F4/80^−^), P2 (MHC II^+^ CD11c^+^ CD11b^+^ F4/80^−^), and P3 (MHC II^+^ CD11c^+^ CD11b^+^ F4/80^+^).

### 4.5. Statistical Analysis

Each treatment group was compared individually to the control group using an unpaired Student’s *t*-test. This approach was chosen to evaluate specific differences between the control and experimental groups without conducting direct comparisons between the experimental groups themselves. Outlier values were identified using the interquartile range (IQR) method. Values falling 1.5 times outside the IQR were excluded from the analysis to ensure the consistency and reliability of the results. Data are presented as mean ± standard error of the mean (SEM), and *p*-values < 0.05 were considered statistically significant.

## 5. Conclusions

In this study, we conducted an exploration of the effects of colony growth factors present in GM-CSF and SL-929 on the differentiation process of bone marrow precursor cells. Our findings highlight a remarkable and significant increase in the percentage of differentiated cells across all three target populations upon GM-CSF stimulation. Notably, 5 ng/mL and 10 ng/mL concentrations of GM-CSF proved notably effective in increasing the percentage of cells and surface protein levels. Our results showed GM-CSF as the superior choice for the in vitro differentiation of APC cells, particularly when high cell yields are required for effective immunotherapeutic interventions. Furthermore, our study unveiled a noteworthy prominence of P3 (CD11c^+^, MHC II^+^, CD11b^+^, and F4/80^+^ cells), which exhibited a substantial response to GM-CSF stimulation. This effect was most pronounced at a concentration of 5 ng/mL, suggesting the potential for a distinct differentiation profile. Our observations contribute to advancing our understanding of the impacts of GM-CSF and SL-929 on cellular differentiation. Moreover, they offer insights into the potential applications of these findings in the context of cell-based immunotherapy and in exploring the roles of cell populations in various diseases. As a next step, further investigations may delve into the underlying mechanisms driving these differentiation responses and their potential therapeutic implications.

## Data Availability

The data related to this study have already been deposited in the Research Data Repository of UNICAMP and is publicly accessible through the following DOI: https://doi.org/10.25824/redu/Y7SBUH.
